# On the Comparison of Group Performance with Categorical Data

**DOI:** 10.1371/journal.pone.0084784

**Published:** 2013-12-31

**Authors:** Carmen Herrero, Antonio Villar

**Affiliations:** 1 University of Alicante & Ivie, Alicante, Spain; 2 Pablo de Olavide University (Seville) & Ivie, Seville, Spain; Universidad Veracruzana, Mexico

## Abstract

There are many different evaluation problems that involve several groups (societies, firms or institutions) whose members can be classified into ordered categories, pursuant to their characteristics or their achievements. This paper addresses these types of problems and provides an evaluation criterion based on the distribution of the agents across categories. The starting point is that of dominance relations in pair-wise comparisons. We say that group *i* dominates group *j* when the expected category of a member of *i* is higher than the expected category of a member of *j*. We introduce the notion of relative advantage of a group to extend this principle to multi-group comparisons and show that there is a unique evaluation function that ranks all groups consistently in terms of this criterion. This function associates to each evaluation problem the (unique) dominant eigenvector of a matrix whose entries describe the dominance relations between groups in pair-wise comparisons. The working of the model is illustrated by means of three different applications.

## Introduction

*Judge a man by the reputation of his enemies*

*(Arabian proverb)*

This paper seeks to present a method to evaluate the relative performance of a given number of groups when the traits or achievements of their members are described by ordered categorical data. Groups can refer to population subgroups, state members of a federation, regions of a country, plants of a firm, etc. Categories may include age intervals, income brackets, health statuses, educational achievements, prestige positions, satisfaction levels, etc. The setting consists, therefore, of a finite set of groups whose members are classified into a given number of ordered categories, which summarize their characteristics or their realizations.

There are two key points in our approach worth stressing. First, we are concerned with the *relative* evaluation of the different groups (i.e. comparing the performance of each group with respect to the others). This, implicitly, means that groups are not unrelated (e.g. they can be regarded as subsets of a larger set). Second, we assume that categories are linearly ordered so that one category can be unambiguously said to precede, be higher than, or be better than another. Our goal is to find a suitable measure of relative group performance taking into account the distribution of group members along the different categories. The evaluation will focus, therefore, on the frequency distribution of the agents in the different cells that arise from the double partition into groups and categories.

An example, among the many real life situations that fits into this scheme, is that of a firm willing to assess the comparative performance of different branches based on the satisfaction levels reported by their clients (e.g. a hotel chain). The groups here are the different branches of the firm, and the categories are the different satisfaction levels, ranging from “fully satisfied” to “not satisfied at all”. The relevant information refers to the distribution of the clients of each branch by levels of satisfaction.

The evaluation of this type of problem requires a criterion to be devised that is capable of dealing with qualitative data, which is the key subject of this paper. Our approach is related to the statistical analysis regarding the similarity between rank distributions and the sociological and economic literature on segregation (see [1], [2], [3], [4], [5]). In these situations, as is also the case with the Lorenz dominance criterion in the inequality literature, we may well find that not all groups are comparable and, as a consequence, that only partial orderings emerge.

We here propose a criterion that allows the relative performance of any (finite) number of groups to be evaluated, using categorical data, in a complete and transitive way. This criterion can be regarded as an extension of the ideas in [6], in the sense that the value we attach to a given group is related to the likelihood of an agent of that group being in a higher position than an agent of any other one. Such an extension is not trivial as it requires the *direct and indirect* relationships between all the groups involved to be taken into account.

Our evaluation function is presented in a constructive way in Section 2.1 by means of three steps. First, we define the relative advantage of group *i* with respect to group *j*, as the ratio between the probability of *i* dominating *j* and the sum of the probabilities of group *i* being dominated by other groups. Second, we obtain the overall advantage of a group as a weighted average of its relative advantages with respect to all other groups. And third, we select an invariant weighting system so that the weights used for that average correspond to those yielded by the overall evaluation function. The value so obtained is called the *worth* of a group.

The evaluation so generated corresponds to the eigenvector of a suitably constructed matrix that incorporates the information on the distribution of the population in the groups across the different categories. This solution has a similar flavour to some of the ways of evaluating the impact of scientific journals (see [7], [8], [9], [10], [11], [12]), as well as with the score allocation in tournaments ([13], [14], [15], [16]).

Section 2.2 provides three examples that illustrate the way in which this evaluation procedure works. The first one refers to the evaluation of Human Capital, in different European countries, that emerges from the distribution of the educational attainments of the working age population. The second one uses the results in the 2013 assessment of cognitive abilities of the adult population, derived from the OECD’s Program for International Assessment of Adult Competence survey (PIAAC), in reading literacy. Finally, we consider the evaluation of health in the former European Union (EU 15), out of the 2011 Eurostat survey on self-perceived health status. Those examples show that the evaluation function can deal with primary data of different nature (subjective or objective, quantitative or qualitative).

A short discussion closes the paper.

## Methods and Results

### 2.1. Theory

Consider a set of g nonempty groups,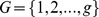, with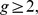 and let 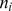 denote the number of members of group 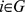. We assume that the individual characteristics of the group members induce a partition in terms of categorical positions 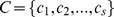, ordered from best to worst, 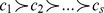. We denote by 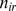 the number of members of group *i* in category *r*. By definition, 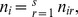 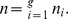 An *evaluation problem* in this context refers to the comparison of the *relative performance* of the different groups in terms of the distribution of their members in the different categories.

Let *a_ir_* denote the share of members of group *i* who belong to category *r*, i.e. *a_ir_* = *n_ir/_n_i_*, and let *p_ij_* denote the *probability of a member of group i being in a higher position than a member of group j.* Since categories are ordered, this probability can be easily computed through the following formula:
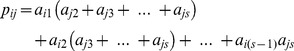
(1)


Similarly, 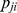 denotes the probability of a representative member of group *j* being at a higher position than a representative member of group *i*. And, consequently, 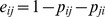 stands for the probability of a member from *i*, picked at random, being at the same position as a member from *j.*

***Remark 1:*** *We shall assume, for the sake of simplicity, that all 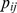 are strictly positive. This point will be discussed later on.*

Consider now the following:

**Definition 1:** *We say that group i dominates group j in a pair-wise comparison when the probability that an individual in group i is at a higher position than an individual in j is larger than the other way around. That is, 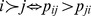.*

This is a sound criterion when there are only two groups involved as it allows their relative performance to be evaluated in an unambiguous way. This type of pair-wise comparison is reminiscent of Lieberson’s *Index of Net Difference* [6]. Extending this principle to a more general setting, involving any finite number of groups, requires some additional elaboration, since pair-wise domination fails to satisfy transitivity. We have to devise a way of comparing the relative position of members in each group with respect to all other groups, taking both direct and indirect relationships into account.

Let *P* denote the set of all pair-wise comparisons in probability terms, 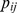, 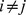. This set fully describes the relevant data of our problem (under the implicit assumption that the size of the groups is immaterial for the evaluation). We shall refer to *P* as a *reduced evaluation problem*, or simply as a *problem*. Consider now the following definitions.

**Definition 2:** *Given a problem P, the relative advantage of group i with respect to group j,* 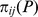 *, is given by:*

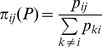
(2)


That is, the relative advantage of group *i* with respect to group *j*, 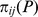, is the ratio between the probability of group *i* dominating group *j*, in a pair-wise comparison, and the sum of the probabilities of group *i* being dominated by any other group. For the family of problems involving only two groups, we have 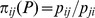, so that 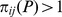 indicates that *i* dominates *j*. Moreover, we find that, for all pair-wise comparisons with any given number of groups, 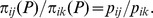 That is, the ratio of the relative advantage of *i* with respect to *j* and with respect to *k* coincides with the ratio of their associated domination probabilities. This is not the case in general, as the probability of being dominated by some other group changes from one to another.

If 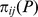 is the relative advantage of group *i* with respect to group *j*, what can we say about the *overall performance* of group *i* ? The simplest way of achieving such a global evaluation is by assigning to each group a weighted average of its relative advantages. That is,
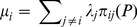
(3)Where 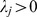 is a measure of the importance attached to group *j* and 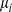 is the resulting overall evaluation of group *i*. The natural question is whether we can find an *invariant* system of weights. That is, a way of attaching the relevance of the different groups, 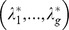, so that: 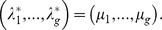 This property would ensure a consistent evaluation, in the sense that the importance attached to the different groups derives, precisely, from the importance that the evaluation function yields.

We refer to such a system of weights as the worth vector, and define the following:

**Definition** **3:** *A consistent evaluation function is a mapping F such that, for each evaluation problem P, it associates its worth to each group. That is, for any problem P we have* 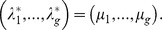 *, with:*

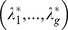
(4)


Note that the worth of a group is higher, all other things being equal, the higher the worth of the groups it dominates.

The case in which there are only two groups involved has an interesting property: the ratio between their worth components coincides with the ratio of the probability of one dominating the other. That is,
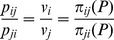



We next show that such a system of weights always exists and it is unique, once the scale has been chosen (and bearing in mind Remark 1). We set the scale so that the average value of the vector components is equal to 1 in order to facilitate the interpretation.

**Theorem 1:** *Let P be an evaluation problem regarding* 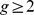 *groups whose members are classified into s ordered categories. A unique consistent evaluation function F exists, with* 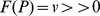,
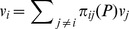
, and 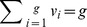.

**Proof:** Consider now that the information relating to problem *P* is arranged in the form of a matrix 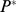 whose 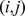 entry is 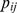, for 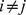, and the diagonal elements of that matrix are given by 
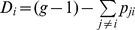


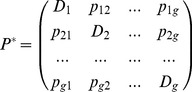
(5)


Matrix P* is simply a particular way of arranging the information concerning the problem under consideration. Now observe that P* is a square matrix with positive entries (i.e. a Perron matrix). Moreover, by construction, all columns of P* add up to 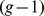. Therefore, P* has a unique dominant positive eigenvalue, equal to 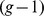, that has associated a strictly positive eigenvector 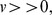 with:
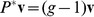
(6)


This eigenvector, 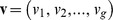, is unique up to a scalar multiplication, so that we can assume, without loss of generality, that 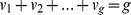. Observe that the *i*th entry of that eigenvector can be written as:
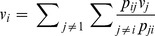
(7)


The evaluation function is thus implicitly defined as follows:
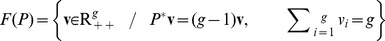
and we obtain the desired result. **Q.e.d.**

Theorem 1 tells us that there is a unique consistent evaluation function *F* that attaches its *worth* to each group. The worth of a group refers to the situation of a representative member *vis a vis* the representative members of all other groups. Function *F* allows the relative advantage of any two groups to be consistently compared: 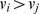 means that the members of *i* are, on average, in a better position than the members of *j*. The components of the worth vector can be interpreted as the limit procedure of the following contest: Take two groups at random, select one individual within each group, and compare them. The group to which the individual with a higher category belongs is selected to keep running the contest and a new individual of this group is randomly selected. This individual will be confronted with another one, selected at random from other group, also chosen at random. We then apply the same principle as before regarding the group that is selected for the next round. The components of the worth vector turn out to be proportional to the length of time that each group is kept competing, when we repeat this process indefinitely.

Computing the worth of the different groups is straightforward, as they correspond to the Perron eigenvector of matrix P*. That feature also permits the worth vector to be interpreted as the limit of a dynamic evaluation process in which the value attached to a group is sequentially adjusted by using matrix P*, starting from any arbitrary evaluation of the different groups. That is, let us take an initial evaluation vector, ω (e.g, 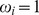 so that the initial evaluation of a group corresponds to the arithmetic mean of its relative advantages), and proceed as follows: 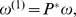 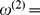 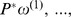 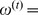 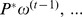 We thus obtain: 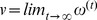.

***Remark 2:*** *We have devised a free access and friendly algorithm to compute the* ***worth vector*** out of the matrix of relative frequencies, which can be directly operated from data in the format of an Excel spreadsheet. The algorithm first constructs the P* matrix (as an internal operation) and then computes the worth vector, suitably normalized. See http://www.ivie.es/valoracion/index.php.

### 2.2. The Working of the Worth Vector: Three Examples

This section is devoted to illustrating how the worth vector works in three different scenarios that involve qualitative and quantitative characteristics as well as subjective and objective data. The first example refers to the composition of Human Capital in different European countries, taken to be the distribution of the working population across educational attainments. Information is here objective and characteristics are purely qualitative. The second illustration refers to the evaluation of cognitive abilities of adult population in some OECD countries, using the data of the 2013 PIAAC with respect to reading literacy. We also find here a case with objective information but, contrary to the first example, the characteristics are defined by intervals of quantitative data. Finally, we provide a comparative evaluation of the perceived health situation of the EU15 countries, taken from the Self-reported Health Status Survey. In this case, inputs are subjective perceptions and categories are purely ordinal. In all three examples, the worth vector provides a complete ranking of the respective countries, according to their relative performance or achievements. As this exercise is merely for illustrative purposes, we shall not delve into the nature of the resulting differences. Yet, in the second and third examples, we provide alternative evaluations in order to enable the outcome of this procedure to be compared.

#### 2.2.1. Comparing the quality of human capital in europe

Human Capital is one of the key determinants of human development and long-term economic growth (see for instance [17] and the references provided there). Countries display a wide variety of educational structures, regarding the qualification of their labour forces, even in relatively homogenous environments, as the 2010 Eurostat data show. Such diversity highlights the difficulty of attaining an overall comparative measure of Human Capital. Our model can be fruitfully applied to this context, in order to obtain a sensible evaluation of the relative quality of Human Capital under the assumption that higher levels of education are preferable.

We compare here the composition of Human Capital in Europe in 2010, in terms of the distribution of the population aged between 25 and 64 years old across the different educational levels, as defined by the International Standard Classification of Education (ISCED). We compare the relative educational achievements of 30 European countries using Eurostat data. Table 1 below describes, in its first three columns, the distribution of the population of the different countries at three educational levels: primary studies (ISCED 0–2), secondary studies (ISCED 3–4), and tertiary studies (ISCED 5–6). The table shows the wide diversity in the Human Capital structure mentioned above. Roughly speaking, Europe presents, on average, a distribution where one half of the adult population has secondary studies, whereas one quarter has primary studies and the remaining quarter tertiary studies. The extreme values for tertiary studies correspond to Finland (37.6%) and, somewhat unexpectedly, to Italy (14.8%). The extreme values for primary studies correspond to Lithuania (8%) and Malta (70.3%). Secondary studies range between 75.2% (Czech Republic) and 16.5% (Portugal). The corresponding coefficients of variation are 0.3 for tertiary studies, 0.3 for secondary studies and 0.6 for primary studies.

**Table 1:** Shares of the population by levels of study and worth vector.

Countries	Primary	Secondary	Tertiary	Worth vector
Austria	0.1623	0.6448	0.1929	**0.869**
Belgium	0.2950	0.3551	0.3499	**0.979**
Bulgaria	0.2077	0.5608	0.2315	**0.871**
Cyprus	0.2501	0.3940	0.3558	**1.095**
Czech Republic	0.0806	0.7518	0.1677	**0.986**
Denmark	0.2212	0.4367	0.3421	**1.130**
Estonia	0.1088	0.5382	0.3529	**1.530**
Finland	0.1697	0.4548	0.3755	**1.388**
France	0.2916	0.4173	0.2912	**0.850**
Germany	0.1371	0.5982	0.2647	**1.126**
Greece	0.3481	0.4128	0.2391	**0.657**
Hungary	0.1871	0.6117	0.2012	**0.839**
Iceland	0.2777	0.3969	0.3254	**0.956**
Ireland	0.2611	0.3658	0.3730	**1.115**
Italy	0.4426	0.4094	0.1480	**0.409**
Latvia	0.1153	0.6159	0.2689	**1.203**
Lithuania	0.0798	0.5938	0.3264	**1.542**
Luxemburg	0.1751	0.4713	0.3537	**1.296**
Malta	0.7028	0.1591	0.1381	**0.222**
Netherlands	0.2703	0.4046	0.3251	**0.970**
Norway	0.1899	0.4461	0.3640	**1.284**
Poland	0.1134	0.6580	0.2286	**1.081**
Portugal	0.6808	0.1647	0.1545	**0.250**
Romania	0.2571	0.6050	0.1380	**0.594**
Slovakia	0.0904	0.7364	0.1732	**0.978**
Slovenia	0.1669	0.5959	0.2372	**0.973**
Spain	0.4689	0.2251	0.3060	**0.619**
Sweden	0.1351	0.5230	0.3418	**1.388**
Switzerland	0.1244	0.5250	0.3506	**1.460**
United Kingdom	0.1487	0.5097	0.3416	**1.341**
UE-27	0.2579	0.4844	0.2577	

Source: Eurostat.

The last column of Table 1 provides the evaluation of the relative quality of Human Capital in those countries according to our evaluation formula (the normalized eigenvector of the associated P* matrix). The worth vector shows that Lithuania, Estonia, Switzerland, Finland, Sweden, and the United Kingdom are the countries with relatively better Human Capital structure, whereas Malta, Portugal, Italy, Romania, Spain and Greece are at the other end of the quality distribution. The coefficient of variation of the worth vector takes on the value 0.35, which is rather large.

#### 2.2.2. Assessment of cognitive abilities from the PIAAC

The *Programme for the International Assessment of Adult Competences* (PIAAC), coordinated by the OECD, is a new database on the cognitive skills of the population between 16 and 65 years old. It provides information on the abilities that people actually have rather than on their formal education. It is, therefore, a valuable complement of the studies carried out on the levels of competence of young people in different fields and for different ages (e.g. the Program for International Students Assessment, PISA). The PIAAC provides cross-section data on the skills of the adult population in the areas of *reading literacy and mathematics.* Twenty-three countries participated in this first wave and a few more will be incorporated in an extension planned for the following three years. The skills assessment is performed using questionnaires and the valuations are measured on a scale of 0–500 points.

PIAAC defines six ***levels of competence***, parameterized by certain thresholds of the test scores (see Table 2). Note that setting of the levels is essentially a qualitative exercise (i.e. the levels are defined in terms of the tasks that individuals are able to perform) even though the resulting categories are made operational through a convenient parameterization.

**Table 2:** Thresholds of the tests scores.

Reading literacy	Range of score points
*Level 5*	>375
*Level 4*	326–375
*Level 3*	276–325
*Level 2*	226–275
*Level 1*	176–225
*Level <1*	<176

Table 3 presents a comparison between the (normalized) worth vector and the mean values. Focusing on the worth vector, we observe that the Netherlands, Sweden and Norway are the European countries well above the mean worth (with Japan, the top country by far). At the end of the ranking, we find Poland, Ireland, France, Spain and Italy, with values between 80% and 46% of the mean worth.

**Table 3:** Distribution of the population by levels of competence and normalized scores and worth vector for reading literacy (2012).

National entities	Level 5	Level 4	Level 3	Level 2	Level 1	Level <1	Worth vector	Mean scores
Australia	0.0133	0.1600	0.4013	0.2972	0.0961	0.0316	**1.262**	**1.022**
Austria	0.0027	0.0830	0.3798	0.3786	0.1307	0.0255	**0.849**	**0.983**
Canada	0.0095	0.1291	0.3763	0.3200	0.1268	0.0383	**1.004**	**1.004**
Czech Republic	0.0040	0.0831	0.4174	0.3774	0.1033	0.0151	**0.976**	**1.008**
Denmark	0.0038	0.0967	0.4010	0.3411	0.1194	0.0382	**0.930**	**0.998**
England (UK)	0.0078	0.1259	0.3646	0.3358	0.1326	0.0335	**0.964**	**0.998**
Estonia	0.0077	0.1101	0.4076	0.3441	0.1105	0.0201	**1.050**	**1.017**
Finland	0.0076	0.1097	0.4060	0.3427	0.1100	0.0200	**1.050**	**1.062**
France	0.0029	0.0748	0.3434	0.3620	0.1638	0.0535	**0.699**	**0.963**
Germany	0.0049	0.1032	0.3698	0.3445	0.1446	0.0335	**0.874**	**0.987**
Ireland	0.0039	0.0815	0.3621	0.3775	0.1322	0.0432	**0.791**	**0.982**
Italy	0.0006	0.0329	0.2654	0.4229	0.2232	0.0554	**0.458**	**0.921**
Japan	0.0120	0.2163	0.4915	0.2305	0.0436	0.0061	**2.167**	**1.085**
Korea	0.0022	0.0792	0.4184	0.3715	0.1067	0.0221	**0.941**	**1.005**
Netherlands	0.0134	0.1722	0.4242	0.2703	0.0932	0.0266	**1.402**	**1.033**
Norway	0.0062	0.1338	0.4256	0.3086	0.0949	0.0307	**1.182**	**1.013**
Poland	0.0066	0.0900	0.3503	0.3654	0.1483	0.0390	**0.795**	**0.986**
Slovak Republic	0.0015	0.0732	0.4455	0.3634	0.0977	0.0191	**0.990**	**1.010**
Spain	0.0015	0.0466	0.2805	0.3946	0.2044	0.0726	**0.502**	**0.926**
Sweden	0.0120	0.1488	0.4157	0.2908	0.0958	0.0370	**1.232**	**1.032**
United States	0.0066	0.1136	0.3572	0.3398	0.1416	0.0407	**0.883**	**0.968**

Source: OECD (PIAAC survey).

The most striking feature of previous comparisons is the huge difference in their variability. The coefficient o variation of the worth vector is more than nine times that of mean scores, in spite of the high correlation between both vectors (around 0.88). Moreover, there are some changes in the ranking between both evaluations, even though they are not many.

#### 2.2.3. Perceived health in the EU 15

We now present an application of the model to the 2011 health comparison of European countries. The data, provided by Eurostat, involve fifteen countries (the EU15) and are from a survey in which people report their perceived state of health, selecting one of the five possible states: Very good, Good, Fair, Bad, and Very bad.

In order to evaluate the health level of a society using those data and to compare the situation of different countries, analysts are bound to attach some cardinal values to those health categories, either by a naive procedure (a “1 to 5” scale) or by means of more sophisticated methods ([18], [19], [20]). Be that as it may, the results so obtained depend on the chosen cardinalization, whose rationale is not always clear. Indeed, several authors have proposed ways out of that difficulty. In [21] apply a family of inequality indices due to [22] that have suitable invariant properties with respect to the cardinalization. [23] deals with the same problem from a different perspective, using socioeconomic variables to order distributions (Lorenz dominance criteria applied on an income-health matrix).

Our model provides an endogenous cardinalization that stems from the distribution of the population between the different health states, without having to decide how much “good health” is better than “bad health”, and so on. Table 4 below describes the distribution of the population between the different states and the evaluation obtained through the worth vector. We have also included the evaluation according to the “1 to 5” scale (normalised as before) in order to obtain a comparative view of the worth vector. This scale gives one point to the “Very bad” health status, two points to the “Bad” state, etc., up to five points to the “Very good” state.

**Table 4:** Distribution of the population within EU 15 by health states in 2011.

Countries	Very good	Good	Fair	Bad	Very bad	Worth vector	“1 to 5” scale
Austria	0.312	0.382	0.215	0.072	0.019	**0.975**	**1.004**
Belgium	0.296	0.439	0.169	0.074	0.021	**1.016**	**1.008**
Denmark	0.280	0.428	0.209	0.058	0.025	**0.940**	**0.999**
Finland	0.216	0.473	0.237	0.063	0.012	**0.796**	**0.984**
France	0.226	0.450	0.236	0.076	0.012	**0.788**	**0.979**
Germany	0.166	0.482	0.270	0.066	0.015	**0.659**	**0.957**
Greece	0.506	0.258	0.146	0.063	0.027	**1.660**	**1.070**
Ireland	0.425	0.401	0.145	0.025	0.005	**1.707**	**1.087**
Italy	0.131	0.516	0.222	0.102	0.030	**0.580**	**0.932**
Luxembourg	0.260	0.465	0.196	0.064	0.015	**0.935**	**1.002**
Netherlands	0.211	0.552	0.179	0.049	0.008	**0.923**	**1.006**
Portugal	0.094	0.403	0.322	0.131	0.049	**0.389**	**0.865**
Spain	0.215	0.536	0.174	0.055	0.020	**0.893**	**0.997**
Sweden	0.385	0.414	0.154	0.037	0.010	**1.453**	**1.063**
UnitedKingdom	0.355	0.420	0.168	0.047	0.010	**1.286**	**1.047**

Worth vector and evaluation with “1 to 5” scale (mean = 1 in both cases).

Source: Eurostat.

The top performers according to the worth vector are Ireland, Greece and Sweden, with values above 40% of the mean worth. At the other extreme we find Germany, Italy and Portugal, with values well below 70% of the mean worth.

Both evaluations show a salient feature: health perceptions are widely different among the citizens of the European Countries, with no correlation whatsoever between self-assessed health and a standard measure of health such as life expectancy at birth (the coefficient of correlation is just 0.0161 for the worth vector and 0.076 for the “1 to 5” evaluation). Note that, even though both procedures yield very similar rankings (and also show a high level of linear correlation, with a coefficient of 0.95), the worth vector discriminates much more between countries. The coefficient of variation of the worth vector is 0.378, almost seven times that of the other evaluation (with a CV of 0.056).

## Discussion

There are many different evaluation problems that involve several groups (societies or institutions) whose members can be classified into ordered categories, pursuant to their characteristics or their achievements. The solution proposed here exploits the information on the distribution of agents across categories in order to provide an estimate of their relative situation. The comparison criterion that lies behind our evaluation method corresponds to the likelihood of an agent in a given group belonging to a higher category than another agent picked at random in any other group.

Our contribution consists of framing the evaluation problem in such a way that the solution to the evaluation corresponds to the dominant eigenvector of a Perron matrix. As a consequence, the solution obtained (the *worth vector*) exhibits simple, useful and well-known properties: existence, uniqueness, positiveness, stability, easy calculation and regular behaviour regarding changes in the parameters.

The *worth vector* not only provides a complete ranking of any given number of groups, but also an endogenous cardinalization that allows a quantitative estimate of their differences. Those features derive from the design of the evaluation formula, which processes the information on all pair-wise dominance probabilities in an integrated way. Interestingly enough, the ranking so obtained agrees with the partial ordering generated by stochastic dominance relationships, as in [22] or [23].

The uniqueness and strict positiveness of the worth vector in Theorem 1 is ensured by the assumption 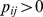, for all 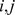. Indeed, this same result can be guaranteed by merely assuming a much weaker condition, namely, the irreducibility of matrix P*. That is, assuming that there is no partition of the set of groups *G* into two disjoint subsets, 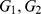, so that 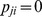, for all 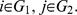 If such a partition exists, i.e., if matrix P* is reducible, it simply means that any group in 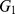 fully dominates any group in 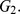 In that case, the valuation vector associated to the dominated groups (those in 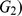 could be zero, and we can only guarantee the strict positiveness for the valuation of groups in 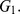 The extreme case of this situation appears when there is a single group dominated by all other groups. In such a case, the dominated group can be regarded as a *dummy,* in the sense that its worth is zero and, moreover, the relative valuation of the remaining groups does not change when we remove the unanimously dominated group. If matrix P* is reducible, then groups in 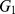 and in 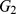 belong to “different classes” and comparing the relative performance within each class is what makes sense. In a similar vein, in [14] only tournament matrices that are irreducible are considered, interpreting that the reducibility of matrices implies a division in different equivalence classes and it is only meaningful to compare elements within the same class.

It is also interesting to point out that the evaluation function in Theorem 1 satisfies a number of standard properties that reinforce its operational and normative appeal. We mention here: (i) *Anonymity:* the evaluation only depends on the characteristics of the groups and not on other aspects such as labels or names; (ii) *Symmetry:* when 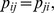 for all pairs of groups, *i*, *j*, then all groups are given identical values; (iii) *Monotonicity:* when some members of a group shift to a higher category, with everything else unchanged, then the evaluation of this group increases; (iv) *Stochastic dominance*: when one group stochastically dominates another one, then, it obtains a higher evaluation (in [22] we find a partial ordering based on this criterion robust to changes in the cardinalization); (v) *Reciprocity*: for those problems involving two groups the relative valuation of the groups coincides with the ratio of the corresponding domination probabilities (see also [14]); and (vi) *Group replication invariance*: the evaluation of the groups depends on the shares of their agents in the different categories but not on the size of those groups.

Note that the worth vector, as any eigenvector, has a degree of freedom, which allows the scale to be fixed at will. We have here chosen a normalization such that the mean value of the worth vector is equal to one. We can thus easily identify the groups that are above or below the mean.

The examples in Section 2.2 show that the procedure can be applied to different types of problems, involving quantitative or qualitative data of an objective or subjective nature. When data are ordinal (examples 2.2.1 and 2.2.3) categories are defined in terms of some property on which there is usually a wide consensus. When data are cardinal (example 2.2.2) the building of the categories usually requires parameterizing some property in terms of the range of values of the outcome variables (as with the levels of competence in the PIAAC study). It is important in that case to avoid the arbitrariness of the categories to ensure the relevance of the evaluation.

The examples in sections 2.2.2 and 2.2.3 include an alternative evaluation of the problem under consideration. This is done in order to better illustrate the nature of the worth vector. By using a common scale we can compare individual values and likewise the spread of the resulting evaluations. We have found a high correlation between both types of evaluation in the two examples, even though there may be some changes in the ranking. Yet the worth vector discriminates much more (the coefficient of variation is much larger). The reason is that those alternative evaluations are obtained for each group independently of the rest (absolute evaluation of individual groups). The worth vector, on the contrary, requires computing the domination relationships of each group with respect to all others. The worth of a group, therefore, depends not only on the distribution of its members on the different categories, but also on the distribution of the agents of all other groups.
